# Integrating Clinical Data and Medical Imaging in Lung Cancer: Feasibility Study Using the Observational Medical Outcomes Partnership Common Data Model Extension

**DOI:** 10.2196/59187

**Published:** 2024-07-12

**Authors:** Hyerim Ji, Seok Kim, Leonard Sunwoo, Sowon Jang, Ho-Young Lee, Sooyoung Yoo

**Affiliations:** 1 Office of eHealth Research and Business Seoul National University Bundang Hospital Seongnam-si Republic of Korea; 2 Department of Health Science and Technology Graduate School of Convergence Science and Technology Seoul National University Seoul Republic of Korea; 3 Department of Radiology Seoul National University Bundang Hospital Seongnam-si Republic of Korea; 4 Department of Nuclear Medicine Seoul National University Bundang Hospital Seongnam-si Republic of Korea

**Keywords:** DICOM, OMOP, CDM, lung cancer, medical imaging, data integration, data quality, Common Data Model, Digital Imaging and Communications in Medicine, Observational Medical Outcomes Partnership

## Abstract

**Background:**

Digital transformation, particularly the integration of medical imaging with clinical data, is vital in personalized medicine. The Observational Medical Outcomes Partnership (OMOP) Common Data Model (CDM) standardizes health data. However, integrating medical imaging remains a challenge.

**Objective:**

This study proposes a method for combining medical imaging data with the OMOP CDM to improve multimodal research.

**Methods:**

Our approach included the analysis and selection of digital imaging and communications in medicine header tags, validation of data formats, and alignment according to the OMOP CDM framework. The Fast Healthcare Interoperability Resources ImagingStudy profile guided our consistency in column naming and definitions. Imaging Common Data Model (I-CDM), constructed using the entity-attribute-value model, facilitates scalable and efficient medical imaging data management. For patients with lung cancer diagnosed between 2010 and 2017, we introduced 4 new tables—IMAGING_STUDY, IMAGING_SERIES, IMAGING_ANNOTATION, and FILEPATH—to standardize various imaging-related data and link to clinical data.

**Results:**

This framework underscores the effectiveness of I-CDM in enhancing our understanding of lung cancer diagnostics and treatment strategies. The implementation of the I-CDM tables enabled the structured organization of a comprehensive data set, including 282,098 IMAGING_STUDY, 5,674,425 IMAGING_SERIES, and 48,536 IMAGING_ANNOTATION records, illustrating the extensive scope and depth of the approach. A scenario-based analysis using actual data from patients with lung cancer underscored the feasibility of our approach. A data quality check applying 44 specific rules confirmed the high integrity of the constructed data set, with all checks successfully passed, underscoring the reliability of our findings.

**Conclusions:**

These findings indicate that I-CDM can improve the integration and analysis of medical imaging and clinical data. By addressing the challenges in data standardization and management, our approach contributes toward enhancing diagnostics and treatment strategies. Future research should expand the application of I-CDM to diverse disease populations and explore its wide-ranging utility for medical conditions.

## Introduction

The accessibility and use of health information in various formats and standards are limited, further limiting the development of advanced data analytics technologies, especially in an era where machine learning and other cutting-edge technologies have become essential for medical research. Integrating these sophisticated analytical tools requires a paradigm shift toward the standardization and harmonization of health care data [1,2]. Standardized data structures are not only beneficial but essential for the effective application of machine learning algorithms as they ensure consistent data quality (DQ), interoperability, and comprehensive analysis across different health care domains. By moving to standardized data formats, we laid the foundation for a more powerful and scalable application of emerging technologies, opening up new possibilities in medical research and patient care.

Several standardization projects and technologies have emerged in response to the demand for integrated approaches [3,4]. Among these, the Observational Medical Outcomes Partnership (OMOP) Common Data Model (CDM) is noteworthy for its advantages in converting diverse sources of data into a consistent format [5,6]. It harmonizes the structure and content of various clinical data sets, facilitating consistent analytical approaches in multi-institutional research. These characteristics ensure high efficiency and accuracy of data interpretation and use, thereby enhancing both the quality and pace of research in the rapidly evolving field of medicine.

Digital Imaging and Communications in Medicine (DICOM) is a universal standard for managing, storing, and transmitting medical images, ensuring interoperability and improved exchange of medical image data and associated information between health care systems. Recent research has focused on the integrated analysis of DICOM and OMOP CDM to promote accessibility to complex medical imaging data and electronic health records [7-11]. These efforts aim to combine detailed imaging metrics with diverse clinical data to contribute to the development of diagnostic and therapeutic strategies through comprehensive data analysis. However, the data duplication problem caused by constructing an instance-level table and the absence of a table that can store annotation data such as labeling (commonly used in image analysis) are major limitations. These limitations must be addressed to effectively manage the complex characteristics of medical imaging data and perform an integrated analysis with clinical information in the OMOP CDM. As the complexity of medical imaging data and the range of DICOM tags increase, effective solutions are required to integrate data seamlessly and consistently.

Lung cancer is the leading cause of cancer-related deaths worldwide and accounts for >20% of all cancer fatalities in South Korea. The etiology, progression, and therapeutic response of lung cancer are intricately linked to a myriad of biological and genetic factors. Therefore, a systematic understanding of the characteristics of lung cancer is paramount for its early detection, prevention, and construction of personalized treatment strategies [12,13]. However, this requires an approach that efficiently integrates high-resolution data across various fields.

In this study, we propose a method to integrate medical imaging data with the OMOP CDM, aimed at enhancing multimodal research capabilities. This approach involves converting DICOM metadata and its annotation data to fit within the OMOP CDM framework and subsequently integrating it into a designed Imaging Common Data Model (I-CDM). We applied this integrated framework to a specific cohort of patients with lung cancer and brain metastases to not only test the feasibility and utility of our approach but also demonstrate its practical application through a series of research scenarios. Additionally, the use of scenarios was intended to showcase use cases that validate the operational functionality of our proposed model within real-world research settings.

## Methods

### Overview

**Figure 1:**
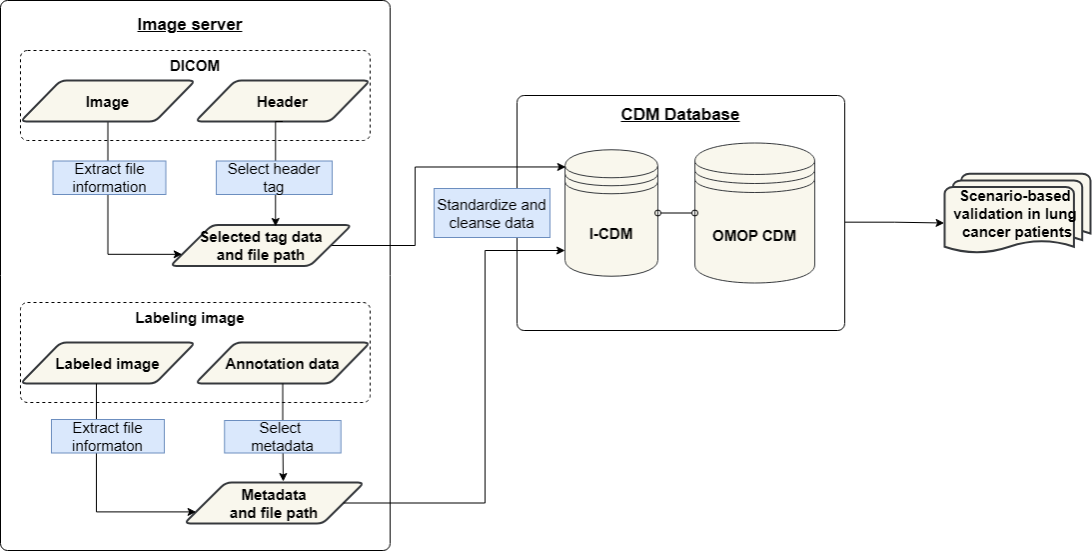
Workflow for I-CDM implementation in lung cancer research. CDM: Common Data Model; DICOM: Digital Imaging and Communications in Medicine; I-CDM: Imaging Common Data Model; OMOP: Observational Medical Outcomes Partnership.

We systematically analyzed and selected the DICOM header tags, verified their data formats, and mapped them to the OMOP CDM framework. To ensure consistency and interoperability in the column naming and definitions, we referenced the Fast Healthcare Interoperability Resources (FHIR) image study profiles, constructed I-CDM tables incorporating an entity-attribute-value (EAV) model for scalability, and performed data preprocessing to maintain data integrity. This allowed us to construct and validate a series of scenarios that combined clinical and imaging information from patients with lung cancer using a structured approach while ensuring interoperability. Figure 1 provides a visual overview of the processes used.

### DICOM Header Analysis

We selected preliminary DICOM header tags that are universally applicable across a spectrum of modalities through a systematic procedure. To ensure the relevance and appropriateness of these tags, we sought consultation with radiology specialists [14-16]. While the DICOM standard provides a framework, it does not enforce a uniform data format. This lack of uniformity has led to variations in data formats across medical institutions and modalities. Accordingly, we validated the extractable data formats, ensuring that only data from nonempty DICOM header tags are extracted to maintain DQ and relevance. For data values where no standard concepts were unavailable, we introduced new custom concepts that are used consistently and comprehensively within the Observational Health Data Sciences and Informatics (OHDSI) framework. Concurrently, we examined the FHIR standard’s ImagingStudy profile of radiological image data to determine the appropriate table and column names [17]. This approach was adopted to ensure that the selected header tags provided comprehensive coverage with respect to established imaging information standards.

### I-CDM Table Modeling

Our database architecture was designed to consolidate a variety of medical imaging data types, including DICOM header tags, study and series-level preprocessing information, and annotation information through labeling. The organization of IMAGING_STUDY, IMAGING_SERIES, IMAGING_ANNOTATION, and FILEPATH tables ensured structured and accessible data collection. The IMAGING_STUDY table refers to the CDM PROCEDURE_OCCURRENCE table associated with the corresponding imaging-order information.

To understand the significance of series-level analysis in medical imaging with reference to the ImagingStudy profile of the FHIR standard, we modeled the IMAGING_SERIES table [18-20] to house values derived from DICOM header tags pertinent to the series. Considering the potential variability in series-specific details owing to distinct imaging equipment or research requirements, we adopted the EAV format. The EAV model provides a data representation framework for the scalable and flexible storage of entities, in which the number and types of attributes (properties and parameters) can vary [21,22]. Using EAV, each attribute-value pair is stored as a separate record, making it easier to populate new data rows without altering the foundational database schema. This model facilitates data expansion without necessitating changes to the foundational table structure.

**Figure 2:**
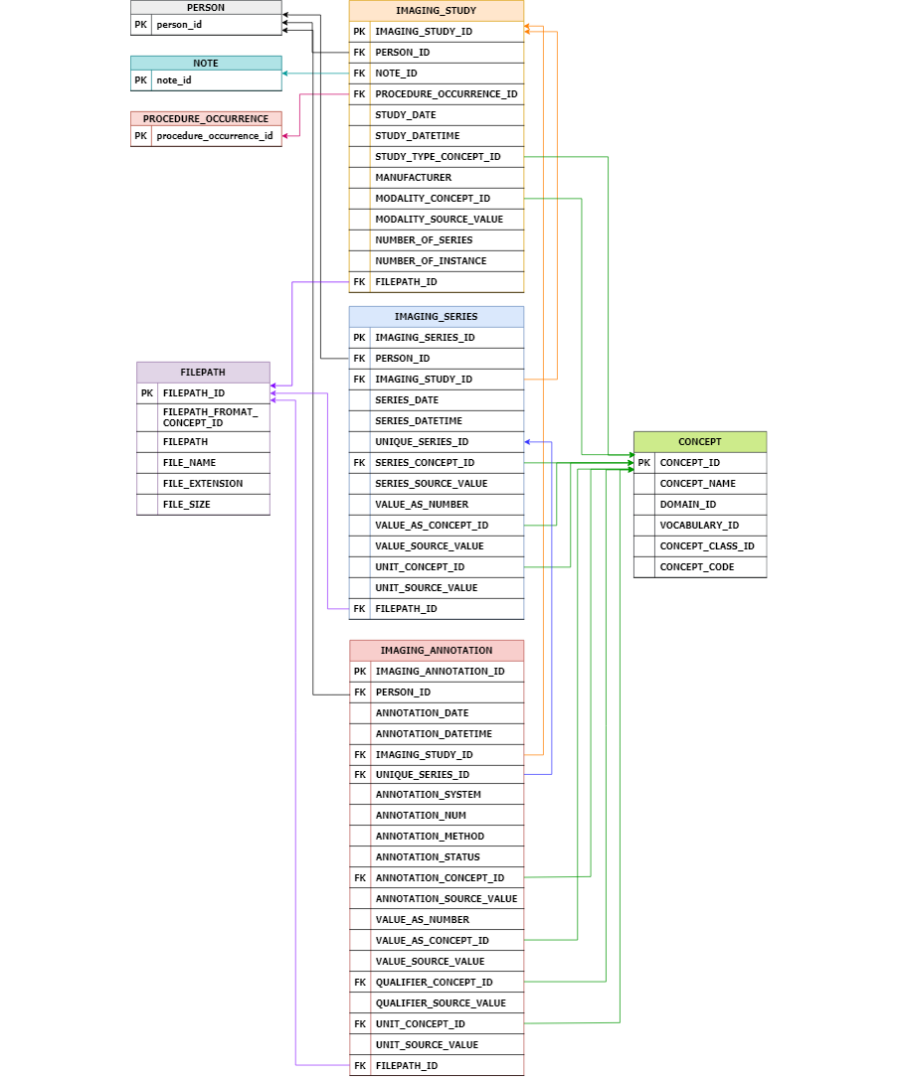
Diagram of Imaging Common Data Model workflow and attributes.

To observe the emergence and ubiquity of automated labeling tools in radiology, we designed an IMAGING_ANNOTATION table [23-26] structured to retain minimal metadata originating from the tools. Similar to the IMAGING_SERIES table, we used an EAV approach to promote the extension of the metadata. Finally, in response to the importance of the file size in image-oriented research and artificial intelligence implementations, we designed a FILEPATH table. This database captured fundamental attributes, such as file size, location, and specific format. Figure 2 shows the diagram constructed according to the I-CDM table definition. In Multimedia Appendix 1, we provide detailed definitions for each column in the IMAGING_STUDY, IMAGING_SERIES, IMAGING_ANNOTATION, and FILEPATH tables for I-CDM. Multimedia Appendix 1 elucidates the data format and captures broadly the attributes of the I-CDM framework. In addition, it specifies whether a column is mandatory, ensuring comprehensive documentation and consistency across data sets.

We analyzed and mapped the categorically constructed columns to the corresponding CDM concept IDs where feasible. The modality information was mapped to the concept IDs of the Procedure class in the CDM specifically related to the imaging equipment. This mapping enabled cross-validation using the method attributes of the linked procedure. The body part category was also aligned with the CDM concept, resulting in terms like “chest” being mapped to the Procedure class as “chest imaging.” For the *IMAGING_ANNOTATION* table, attributes such as the labeling plane and area were harmoniously mapped to the standard CDM concept IDs. In instances where mapping to standard CDM codes proved challenging, custom concepts were designated without limiting the classes and domains. Here, the original data extracted from the images were consistently included in the source-value column to ensure data fidelity. Additionally, in anticipation of RadLex potentially being adopted as a standard vocabulary within the OHDSI framework, we have also suggested additional RadLex mappings for our data set. RadLex, developed by the Radiological Society of North America, is a comprehensive terminology system designed to standardize the names of radiological diagnoses, findings, and procedures. It encompasses a wide range of terms used in medical imaging, making it an invaluable resource for enhancing the comprehensiveness of medical imaging vocabularies within our data set. Multimedia Appendix 2 provides a detailed map of the standard terminology used in our I-CDM with their corresponding OMOP CDM concept IDs.

We expanded our methodological framework by developing a Python-based tool, publicly available on GitHub, for automatic conversion and integration of DICOM files into our I-CDM [27]. This tool was designed to work in conjunction with PostgreSQL to effectively create and populate essential tables such as IMAGING_STUDY, IMAGING_SERIES, and FILEPATH directly from specified DICOM file directories. Notably, the tool includes an algorithm to map the extracted DICOM header data systematically to the corresponding CDM concept IDs. This functionality ensures that the medical image data are not only accurately integrated into the I-CDM but also aligned with the standardized terminologies and classifications of the OMOP CDM. For practical applications, we chose the NSCLC-Radiomics open data set from The Cancer Imaging Archive. The NSCLC-Radiomics data set was used solely and only for the purpose of testing our DICOM file–to-PostgreSQL conversion tool, confirming its functionality with generic DICOM files, and providing a publicly shareable example of the processed output. After reading DICOM files from the NSCLC-Radiomics data set, our tool methodically constructs I-CDM tables within PostgreSQL, thereby streamlining the data integration process.

### Data Preprocessing for I-CDM Table Construction

Before data preprocessing, all personal identifiers were removed to maintain patient confidentiality and protect personal information. Our first step was to ID and categorize the images into a series, ensuring that each series-specific folder contained only pertinent images, thereby maintaining a hierarchical directory structure. The Series Description in a DICOM header often comprises terms and abbreviations that describe the image characteristics. We performed a detailed analysis of the Series Descriptions of selected images to discern imaging attributes, such as the image plane, the presence of enhanced contrast, and designations such as low-dose, T1, or T2, among others. Based on a combination of these criteria, we designed rule-based naming conventions for folders, aiming for descriptive and meaningful names. Furthermore, we extracted information on the presence of “Black Blood” imaging in magnetic resonance imaging (MRI) scans using DICOM header data, which assisted in preparing data for the construction of the imaging series table.

To construct the imaging annotation table, 2 radiology experts identified and labeled the lesions on the chest computed tomography (CT) and brain MRI scans. Subsequently, we extracted metadata related to the labeled regions, such as area dimensions and characteristics.

### Validation of I-CDM for Lung Cancer Studies

#### Overview

This study sought to define a research cohort comprising patients aged 18 years and older with primary lung cancer and structure the metadata of all chest x-ray, chest CT, and brain MRI images using I-CDM. Using the structured data, we aimed to elucidate the unique and major characteristics of patients with lung cancer by analyzing various scenarios.

#### Scenario 1: Association of Hypertension on Imaging Frequency in Patients With Epidermal Growth Factor Receptor Mutation-Positive Lung Cancer Receiving Osimertinib

We investigated the association of hypertension on imaging frequency in patients with lung cancer who were prescribed osimertinib, an epidermal growth factor receptor tyrosine kinase inhibitor. By comparing the frequency of CT imaging between groups with and without hypertension, this study aimed to determine whether the presence of hypertension affects imaging frequency in patients undergoing osimertinib treatment.

#### Scenario 2: Correlation Between Ground-Glass Nodules and Solid Tumor Volume in Lung Cancer

Using annotated data from chest CT scans to compare tumor volumes in patients with lung cancer who have ground-glass nodules (GGNs) with those who have solid nodules, we aimed to explore the relationship between ground-glass opacity nodules and tumor volume in lung cancer.

#### Scenario 3: Use of Low-Dose CT in Diagnostic Imaging for Lung Cancer

This scenario investigates the number of CT series, each consisting of more than 150 image instances, in patients who have undergone low-dose CT for lung cancer diagnosis. This study aimed to evaluate the adequacy of low-dose CT for providing diagnostic information while minimizing radiation exposure in patients.

#### Scenario 4: Number of Enhanced T1-Weighted MRI Images With a Slice Thickness of <1 mm in Patients With Lung Cancer Diagnosed at Younger Than 60 Years

This scenario targets patients with lung cancer diagnosed at younger than 60 years of age and involves quantifying the number of enhanced T1-weighted MRI images with a slice thickness of <1 mm. The collected data highlighted the volume of images available for subsequent annotation, and facilitated an in-depth radiological analysis of patient demographics.

### Ethical Considerations

This study was approved by the Institutional Review Board of Seoul National University Bundang Hospital (approval B-2202-738-004; date: April 14, 2022). All procedures performed in this study involving human participants were in accordance with the ethical standards of the institutional and/or national research committee and with the 1964 Helsinki declaration and its later amendments or comparable ethical standards.

### Statistical Analysis

Statistical analyses were performed using R software (version 4.2.2; The R Foundation for Statistical Computing). Descriptive statistics were used to summarize the data, including the calculation of means for continuous variables and frequency counts for categorical variables. The data were categorized as necessary to facilitate further analysis.

## Results

### Conversion of DICOM Data to the OMOP CDM Format: Realization and Integration in the I-CDM Framework

To systematically organize and efficiently manage the extensive collection of imaging data for the cohort of patients with lung cancer diagnosed between 2010 and 2017, sourced at Seoul National University Bundang Hospital, we structured the I-CDM into 4 basic tables: IMAGING_STUDY, IMAGING_SERIES*,* IMAGING_ANNOTATION, and FILEPATH (Table 1). The data set included imaging data from follow-ups in 2003-2021.

The I-CDM categorized 282,098 IMAGING_STUDY *records*, which were systematically linked to OMOP. This link provides an extensive overview of patient imaging trajectories and clinical data. The database contains 5,674,425 image series encompassing 47,381,027 individual image instances. Of the 282,098 records in the data set, 282,028 records contained information across various modalities, each containing a corresponding “number of series and instance” columns within the database.

**Table 1:** Imaging Common Data Model (I-CDM) data summary for lung cancer cohort (number of tables and records for data from 2003 to 2021 for patients diagnosed with lung cancer from 2010 to 2017).

I-CDM table or column name		Record count, n	Records with data, n (%)	Unique values
**IMAGING_STUDY**		282,098		
	Modality	282,098	282,028 (99.9)	3
	Manufacturer	282,098	281,940 (99.9)	265
	Number of series	282,098	282,098 (100)	35
	Number of instance	282,098	282,098 (100)	1825
**IMAGING_SERIES**		5,674,425		
	Body part examined	382,517	382,517 (100)	25
	Laterality	85,118	85,118 (100)	3
	Slice thickness	411,351	411,351 (100)	1099
	Series description	654,247	635,208 (100)	12,535
	Window center	685,526	685,526 (100)	29,815
	Window width	685,848	685,848 (100)	31,393
	Patient position	458,770	444,770 (100)	11
	Columns	717,154	717,154 (100)	2020
	Rows	717,154	717,154 (100)	2066
	Number of instance	717,169	717,169 (100)	626
	BB^a^/non-BB	12,943	12,943 (100)	2
**IMAGING_ANNOTATION**		48,536		
	Annotation system	48,536	48,536 (100)	5
	Annotation text	3013	2689 (100)	69
	Volume	11,153	11,153 (100)	3298
	Long axis	31,353	31,333 (100)	159
	Surface	3009	3009 (100)	2492
**FILEPATH**		1,000,361		
	File path	1,000,361	1,000,361 (100)	998,844
	File size	1,000,361	1,000,361 (100)	681,026

^a^BB: blood-brain barrier contrast.

The IMAGING_SERIES data represents a testament to the scale and complexity of the data set, with the 5,674,425 series illustrating the vast range of radiological examinations included in this framework. A total of 382,517 records detailing the examined body parts underscored the targeted nature of radiological diagnostics. The data set was also characterized by an array of parameters, with 685,526 records for window centers and 685,848 for window widths. The structural resolution was meticulously captured with 717,154 data points for both the columns and rows, reflecting the intricate images. Each series was contextualized with descriptions recording the purpose and context of the imaging sequence in 654,247 data records. In total, 12,943 images were classified into blood-brain barrier contrast (BB)/non-BB categories to indicate the presence or absence of BB, highlighting the usefulness of specialized imaging sequences for detailed neurovascular assessments.

Table 2 displays the distribution of DICOM data across different modalities for patients with lung cancer, indicating the number of studies and series for each modality. X-ray examinations usually consist of a single series, whereas MRI and CT scans frequently include multiple series per study to accommodate a variety of imaging sequences. This highlights the detailed and complex nature of lung cancer diagnosis and monitoring. The structured categorization within the IMAGING_SERIES table using an EAV model, where the MRI data comprise 11 categories, including the BB/non-BB distinction. This implies a detailed classification of the MRI data, unlike the x-ray data, which are classified into 7 categories representing the variability of the desired parameters for each imaging modality. For x-ray images (n=1,410,844), the features are distributed as follows: number of instances (n=201,755, 14.3%), columns (n=201,755, 14.3%), rows (n=201,755, 14.3%), window width (n=200,938, 14.2%), window center (n=201,755, 14.3%), series description (n=201,755, 14.3%), patient position (n=200,938, 14.2%), slice thickness (n=200,938, 14.2%), body part examined (n=201,755, 14.3%), laterality (n=201,755, 14.3%), and BB_NonBB (n=201,755, 14.3%).

CT images (n=2,982,176) show the following feature distribution: number of instances (n=360,845, 12.1%), columns (n=360,845, 12.1%), rows (n=360,845, 12.1%), window width (n=360,845, 12.1%), window center (n=360,845, 12.1%), series description (n=360,845, 12.1%), patient position (n=360,845, 12.1%), slice thickness (n=360,845, 12.1%), body part examined (n=360,845, 12.1%), laterality (n=286,281, 9.6%), and BB_NonBB (n=116,305, 3.9%).

For MRI images (n=1,281,432), the distribution is: number of instances (n=140,958, 11%), columns (n=140,958, 11%), rows (n=140,958, 11%), window width (n=138,395, 10.8%), window center (n=138,395, 10.8%), series description (n=139,686, 10.9%), patient position (n=142,235, 11.1%), slice thickness (n=142,235, 11.1%), body part examined (n=142,235, 11.1%), laterality (n=80,733, 6.3%), and BB_NonBB (n=91,984, 7.1%).

*The* IMAGING_ANNOTATION table had 48,536 annotations predominantly sourced from CT and MRI scans. These annotations offer a detailed exploration of the examined regions, with volumetric and long-axis measurements documented. This granular level of detail is critical for the precise characterization and monitoring of diseases supported by a comprehensive understanding of the annotated regions. The FILETATH records within the I-CDM table (totaling 1,000,361) served as a bridge between the CDM tables and actual image file paths, spanning approximately 9.6 terabytes of image data. This illustrates not only the substantial volume of image data but also the expansive nature of our image repository within the I-CDM framework. In addition, DICOM folders were organized in a series, and common imaging characteristics were identified through series descriptions to assign meaningful folder names, further streamlining the data structure for efficient management and retrieval.

**Table 2:** Count data based on modality and type.

Modality	Study count	Series count
X-ray	201,569	201,770
CT^a^	65,923	373,116
MRI^b^	14,590	142,254

^a^CT: computed tomography.

^b^MRI: magnetic resonance imaging.

### Validation of I-CDM Scenarios for Enhanced Imaging and Treatment Classification in Patients With Lung Cancer

#### Scenario 1: Hypertension and Imaging Frequency in Patients Treated With Osimertinib

**Figure 3:**
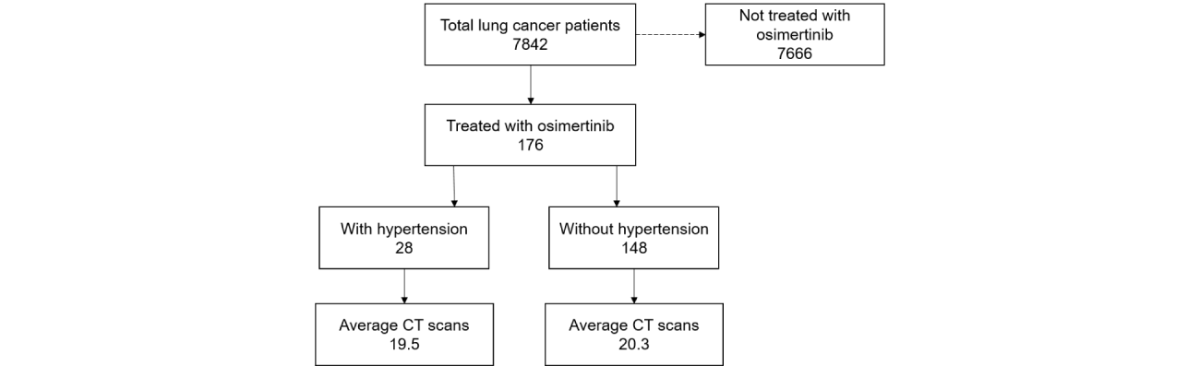
Diagram of CT scan frequency comparison according to hypertension status among osimertinib-treated patients with lung cancer. CT: computed tomography.

Among the total cohort of 7842 patients with lung cancer, 176 (2.24% of the total) prescribed osimertinib were diagnosed with hypertension. In the osimertinib arm, 28 (0.36%) patients had hypertension. The average number of CT scans in patients with and without hypertension was 19.5 and 20.3, respectively, indicating that hypertension did not significantly affect the frequency of CT imaging in patients with lung cancer receiving osimertinib treatment. Figure 3 shows this comparison of CT scan frequency over hypertension status among patients treated with osimertinib.

#### Scenario 2: Nodule Characterization and Volume Measurement in CT Imaging

We evaluated 1947 annotated CT scans from 1929 patients and observed that a significant number of GGNs contained solid components, necessitating labeling of both components within the GGNs. Our comparative analysis focused on the mean volume of solid nodules within GGNs compared with those without GGNs. In total, 673 GGNs were identified in 626 patients, of which 649 were classified as having solid nodules. The average volume of the GGN was 8135.616 mm^3^, whereas the volume of solid nodules within the GGN was 2578.006 mm^3^. In contrast, 1343 solid nodules without GGN were found in 1319 patients, with an average volume of 34,712.58 mm^3^. This corroborates the findings of previous studies indicating that solid nodules, especially those not associated with GGNs, tended to be more abundant [28]. Our results provide additional evidence supporting these observations on the nodal nature of lung pathologies.

#### Scenario 3: Use of Low-Dose CT and Instance Range

Among the 63,446 CT studies conducted in the cohort of patients with lung cancer, which included 2,725,899 series, 48,587 were identified as low-dose imaging studies. Of these, 41,336 included >150 instances. The instance count in the 3 low-dose CT images varied significantly, with the smallest and largest series consisting of three and 633 instances, respectively. This highlights the need for low-dose imaging to capture extensive data while minimizing radiation exposure.

#### Scenario 4: MRI Imaging With 1-mm Thick Slice and T1 Enhancement

In Scenario 4, which focused on MRIs of 1-mm thick slices under T1 enhancement, our analysis of 137,566 MRI series identified 31,851 series using T1-weighting at the specified slice thickness, 5235 of which were associated with patients aged ≤60 years. This scenario allows the preemptive examination of images to be labeled, integrating image patterns and nodule characteristics with clinical data. This approach not only facilitates the identification of the scale of target images to be annotated but also enables precise quantification of the images that meet the specified criteria. Table 3 shows the categorization of the MRI series using I-CDM, providing a visual summary of the data refined by slice thickness and T1 enhancement and further filtered to include patients aged ≤60 years within the studied patient cohort.

**Table 3:** Magnetic resonance imaging series analysis using Imaging Common Data Model.

MRI^a^ series type	Count
Total MRI series	137,566
T1 weighted	74,692
Contrast enhanced	51,582
Slice thickness ≤1 mm	31,851
Age of 60 years or younger	5235

^a^MRI: magnetic resonance imaging.

### I-CDM Data Quality Check

We ensured DQ based on a set of 44 comprehensive DQ rules (outlined in Multimedia Appendix 3), focusing on the Radiation CDM quality assurance framework. These rules encompassed a broad spectrum of checks, including the evaluation of DICOM series and instance counts, data type consistency, and the accuracy of the linkage between the data set and existing clinical tables within the CDM. All data entries successfully met these criteria, indicating compliance with quality standards. Among these rules, those pertaining to interdata relationships and outlier detection were instrumental in validating the integrity of the data set. A selection of these quality checks and their outcomes are presented in Table 4, highlighting the importance of ensuring data quality and integrity within I-CDM.

**Table 4:** Selected data quality assurance rules and outlier analysis results from Multimedia Appendix 3.

CDM_TABLE, CONTCEPT_NAME, and check description			Threshold		Result (%)		Error (n)			
**IMAGING_STUDY**										
	**NUMBER_OF_SERIES**									
		The NUMBER_OF_SERIES must be equal to the number of series in the IMAGING_SERIES table with the same IMAGING_STUDY_ID. This ensures that the number of series recorded in the IMAGING_STUDY matches the actual series entries in the related table		At least 95% match		PASS (99.9)		202		
**NUMBER_OF_INSTANCE**										
		The NUMBER_OF_INSTANCE must equal the sum of VALUE_AS_NUMBER for entries in the IMAGING_SERIES table where SERIES_CONCEPT_ID equals NUMBER_OF_INSTANCE, under the condition that they are mapped between the 2 tables. This is to verify that the number of instances (images) reported in the IMAGING_STUDY corresponds to the aggregated count of instances from the series data		At least 95% match		PASS (99.9)		109		
	**NUMBER_OF_SERIES, NUMBER_OF_INSTANCE**									
		The presence of a Rule of NUMBER_OF_SERIES necessitates the presence of a Rule of NUMBER_OF_INSTANCE.		At least 95% match		PASS (99.9)		1		
**IMAGING_SERIES**										
	**SERIES_CONCEPT_ID = SliceThickness**									
		VALUE_AS_NUMBER must exist and be a numeric value for at least 99% of the records		At least 99% of records must have a nonmissing		PASS (99.8)		496		
	**SERIES_CONCEPT_ID = Rows**									
		VALUE_AS_NUMBER must exist and be a numeric value for at least 99% of the records		At least 99% of records must have a nonmissing		PASS (100)		100		
		Outliers, defined as values beyond the 1st and 99th percentiles, should be reviewed		Outliers should be under 5%		PASS (1.2)		8952		
	**SERIES_CONCEPT_ID = Columns**									
		VALUE_AS_NUMBER must exist and be a numeric value for at least 99% of the records		At least 99% of records must have a nonmissing		PASS (100)		—^a^		
		Outliers, defined as values beyond the 1st and 99th percentiles, should be reviewed		Outliers should be under 5%		PASS (1.0)		7251		
	**SERIES_CONCEPT_ID =** **BB/non-BB**									
		Values must be exclusively “Positive” or “Negative”, ensuring they represent these specific states without including the concept IDs 45884084 and 45878583		100% of records must have as one of the specified valid IDs		PASS (100)		—		
**IMAGING_ANNOTATION**										
	**ANNOTATION_** **CONCEPT_ID** **= Long axis**									
		VALUE_AS_NUMBER must exist and be a numeric value		No missing values for VALUE_AS_NUMBER		PASS (100)		—		
		Outliers, defined as values beyond the 1st and 99th percentiles, should be identified and reviewed to ensure they accurately reflect the intended measurements.		Outliers should be under 5%		PASS (0.2)		77		
	**ANNOTATION_** **CONCEPT_ID** **= Volume**									
		VALUE_AS_NUMBER must exist and be a numeric value		100% of records must be numeric and nonnull		PASS (100)		—		
	**ANNOTATION_** **CONCEPT_ID** **= annotation_text**									
		VALUE_SOURCE_VALUE must contain a nonempty text value		100% of records must be numeric and nonnull		PASS (100)		—		
	**ANNOTATION_** **CONCEPT_ID** **= surface area**									
		VALUE_AS_NUMBER must exist and be a numeric value		100% of records must be numeric and nonnull		PASS (100)		—		

^a^Not applicable.

## Discussion

### Principal Results

This study proposes a method for integrating clinical and imaging data using I-CDM. By converting DICOM data into the OMOP CDM format and integrating it into the I-CDM framework, we implemented a systematic approach to efficiently manage medical imaging data. This approach enabled the connection and analysis of clinical and imaging data in different contexts. Additionally, detailed schema overviews for the use cases of integrating I-CDM can be found in Multimedia Appendix 4, and a comparison between MI-CDM and I-CDM is provided in Multimedia Appendix 5.

### Limitations

The limitation of this study is its lack of consideration for the resources required for processing DICOM images and integrating annotation information. To customize and add data according to researcher needs using the EAV model, comprehensive knowledge and expertise on DICOM standards and tags are required [29]. Furthermore, integrating image annotation data within the I-CDM framework not only demands sufficient resources [30-32], but also requires advanced data management strategies to expand the integration and harmonization of data sets from various imaging modalities beyond chest CT, x-ray, and brain MRI [33,34]. Moreover, focusing exclusively on a cohort of patients with lung cancer narrowed the scope of the study. Additionally, in our study, the scenarios were designed to validate the functionality of the proposed model using actual medical data. These scenarios were deliberately simplified to ensure effective management within the capabilities of the implemented I-CDM framework. Future research will benefit from the incorporation of expanded annotation data, enabling more complex analyses, such as longitudinal comparisons of tumor sizes pre and posttreatment in individual patients.

### Comparison With Prior Work

In this study, diverging from previous Radiology-CDM research, we refined the integration of imaging examination data by linking them with the PROCEDURE_OCCURRENCE table, which enables a more efficient analysis through improved data connectivity. Moreover, unlike previous research that relied on RadLex for standard terminology, this study directly mapped DICOM terms to OMOP CDM standard terminologies. This direct mapping simplifies the process and enables the use of custom codes, thus facilitating a deeper analytical integration of clinical and imaging data. And this study takes a distinct approach compared with recent I-CDM studies [35,36]. Our method enhances the analytical scope by facilitating the storage and management of annotation information. This ensures that imaging-related data, including annotations, can be comprehensively managed within the I-CDM framework. By using the EAV model, our study introduced flexibility in managing various data types and structures, rendering our approach adaptable to evolving research needs and data characteristics. Consequently, it exhibits good flexibility and adaptability, especially in research requiring integrated analysis of clinical and imaging data. Considering the file sizes associated with imaging data, effective file management is essential. Our study used the FILEPATH table to connect I-CDM with the original imaging data, including file extension and size information, to ensure quick access to file details and facilitate efficient management.

### Scalability and Applicability of the I-CDM

The lung cancer cohort in this study was initially used to validate the functionality of the proposed I-CDM tables using actual medical image data. In future studies, the model is not only adaptable to lung cancer but also designed to accommodate a wider spectrum of medical conditions, including various tumor types and cardiovascular diseases. By leveraging OMOP CDM’s standard vocabulary for “modality_concept_id” and “body part examined” (“value_as_concept_id”), the model can be broadly adapted to accommodate various diseases or different settings beyond lung cancer. This adaptability ensures that any additional data items users might require can be seamlessly integrated by aligning with OMOP CDM standard vocabulary, underlining the framework’s potential for broad application across diverse medical data and settings. Furthermore, while RadLex is extensively used in medical imaging vocabularies, it is not yet included as a standard vocabulary in the OHDSI framework. Even if RadLex were incorporated, it would not cover all concepts related to imaging. Therefore, we had to consider various vocabularies to ensure comprehensive coverage. Recognizing this, we aimed to build the I-CDM by maximizing the use of existing OHDSI vocabularies according to OHDSI principles, rather than proposing new vocabularies. We have proactively suggested mapping terms compatible with RadLex within our study wherever possible. In the new scenario, the principle for term selection involves mapping the standard concept to the granularity level of the source data. This is achieved by selecting the term from the standard vocabulary that most accurately represents the clinical meaning. In addition to the features we have currently mapped, our study focused on lung cancer, but for other diseases, there are important concepts in imaging that should be considered. For instance:

- Ultrasound image tags: Commonly used tags include “Transducer Frequency,” “Gain,” and “Depth of Field,” which are critical for analyzing the quality and characteristics of ultrasound images.
- Spine x-ray tags: Relevant tags such as “KVP” (Kilovoltage Peak), “Exposure Time,” and “Focal Spot Size” are essential for understanding the technical parameters that affect image quality.
- Body part imaging concepts: Terms like “Entire Thorax,” “Entire Liver,” and “Entire Pelvis” are crucial for precisely describing the anatomical region being imaged, which can vary significantly depending on the disease or condition being studied.

These examples ensure that the I-CDM framework is adaptable and capable of integrating a wide range of imaging data characteristics and supporting diverse medical conditions and research scenarios.

### Conclusions

This study implemented a systematic approach for the efficient management of medical imaging data, achieving integration of clinical and imaging data through the development of the I-CDM framework and the conversion of DICOM data into the OMOP CDM format. Future efforts should strive to broaden the application of the I-CDM framework to encompass various disease populations and include diverse imaging techniques for different body parts, such as abdominal CT, spine MRI, and liver MRI, thereby enhancing its applicability. Expanding its scope to incorporate these imaging modalities is crucial for conducting more comprehensive investigations into the utility of merging clinical and imaging data across different health conditions.

## Appendix

Multimedia Appendix 1 Imaging Common Data Model table.

Multimedia Appendix 2 Imaging Common Data Model mapping Observational Medical Outcomes Partnership (OMOP) Common Data Model (CDM) concept ID.

Multimedia Appendix 3 Data quality check rule and result.

Multimedia Appendix 4 Use case for integrating Imagining Common Data Model: detailed schema overview.

Multimedia Appendix 5 Comparison of Medical Imaging Common Data Model and Imaging Common Data Model.

## Abbreviations

BB: blood-brain barrier contrast
CDM: Common Data Model
CT: computed tomography
DICOM: Digital Imaging and Communications in Medicine
DQ: data quality
EAV: entity-attribute-value
FHIR: Fast Healthcare Interoperability Resources
GGN: ground-glass nodule
I-CDM: Imaging Common Data Model
MRI: magnetic resonance imaging
OHDSI: Observational Health Data Sciences and Informatics
OMOP: Observational Medical Outcomes Partnership
