# Supplementary material for: Integrating Clinical Data and Medical Imaging in Lung Cancer: Feasibility Study Using the Observational Medical Outcomes Partnership Common Data Model Extension
Source: JMIR Med Inform. 2024 Jul 12;12:e59187. doi: 10.2196/59187 (PMC11282389; doi:10.2196/59187)
Supplement: Multimedia Appendix 1 [file medinform_v12i1e59187_app1.docx]

## Appendix1

Table. IMAGING-CDM Table

| **TABLE** | **CDM Column** | **User Guide** | **DATA_TYPE** | **Required** |
| --- | --- | --- | --- | --- |
| **IMAGING_STUDY** | IMAGING_STUDY_ID | Unique identifier for each imaging study | INT | YES |
|  | PERSON_ID | Foreign key to a Person in the PERSON table | INT | YES |
|  | NOTE_ID | Foreign key to clinical notes in the NOTE table | INT | NO |
|  | PROCEDURE_OCCURRENCE_ID | Foreign key to the associated procedure in PROCEDURE_OCCURRENCE table | INT | YES |
|  | STUDY_DATE | The date when the imaging study was performed | DATE | YES |
|  | STUDY_DATETIME | The date and time when the imaging study was performed | DATETIME | NO |
|  | FILEPATH_ID | Foreign key for the image file path or location | INT | YES |
|  | STUDY_TYPE_CONCEPT_ID | Concept ID representing the type of imaging study | INT | YES |
|  | MANUFACTURER | Name of the imaging device's manufacturer | VARCHAR2 (100) | NO |
|  | MODALITY_CONCEPT_ID | Concept ID representing the modality of the imaging study | INT | YES |
|  | MODALITY_SOURCE_VALUE | The source's specific code or designation for the modality | VARCHAR2 (26) | YES |
|  | NUMBER_OF_SERIES | The total number of series in the imaging study | INT | YES |
|  | NUMBER_OF_INSTANCE | The total number of instances (images) across all series in the imaging study | INT | YES |
| **IMAGING_SERIES** | IMAGING_SERIES_ID | Unique identifier for each imaging series | INT | YES |
|  | PERSON_ID | A foreign key that refers to a unique person in the PERSON table | INT | YES |
|  | FILEPATH_ID | Foreign key for the file path where imaging data is stored | INT | YES |
|  | IMAGING_STUDY_ID | A foreign key to the unique study in the IMAGING_STUDY table | INT | YES |
|  | SERIES_DATE | The date of the imaging series | DATE | YES |
|  | SERIES_DATETIME | The date and time when the imaging series was taken | DATETIME | NO |
|  | UNIQUE_SERIES_ID | A unique identifier for the imaging series across studies | VARCHAR2 (200) | YES |
|  | SERIES_CONCEPT_ID | A key that refers to a standardized concept identifier for the series type in the CONCEPT table | INT | YES |
|  | SERIES_SOURCE_VALUE | The source code for the series as it appears in the source data | VARCHAR2 (100) | YES |
|  | VALUE_AS_NUMBER | A numerical value associated with the series, such as measurement or count | INT | NO |
|  | VALUE_AS_CONCEPT_ID | A key to a concept that reflects the value in the CONCEPT table | INT | NO |
|  | VALUE_SOURCE_VALUE | The source value for the value as it appears in the source data | VARCHAR2 (200) | YES |
|  | UNIT_CONCEPT_ID | A key to a concept that reflects the unit for the value in the CONCEPT table | INT | NO |
|  | UNIT_SOURCE_VALUE | The source code for the unit as it appears in the source data | VARCHAR2 (26) | NO |
| **IMAGING_ANNOTATION** | IMAGING_ANNOTATION_ID | Unique identifier for each imaging annotation | INT | YES |
|  | PERSON_ID | Foreign key to a Person in the PERSON table | INT | YES |
|  | ANNOTATION_DATE | Date when the annotation was recorded | DATE | YES |
|  | ANNOTATION_DATETIME | Date and time when the annotation was recorded | DATETIME | NO |
|  | FILEPATH_ID | Foreign key for the file path where the imaging data is stored | INT | YES |
|  | IMAGING_STUDY_ID | Foreign key to the imaging study in the IMAGING_STUDY table | VARCHAR2 (50) | YES |
|  | UNIQUE_SERIES_ID | Unique identifier for the imaging series across studies | VARCHAR2 (250) | YES |
|  | ANNOTATION_SYSTEM | name of the annotation software or method used | VARCHAR2 (50) | YES |
|  | ANNOTATION_NUM | A unique identifier for each annotation in the study | INT | NO |
|  | ANNOTATION_METHOD | Describes the annotation technique (e.g., automated, manual) | VARCHAR2 (100) | NO |
|  | ANNOTATION_STATUS | Indicates the current progress status of the annotation(e.g.,complete, ongoing) | VARCHAR2 (50) | NO |
|  | ANNOTATION_CONCEPT_ID | Concept ID representing the type of annotation | INT | YES |
|  | ANNOTATION_SOURCE_VALUE | The source's specific code or designation for the annotation | VARCHAR2 (250) | YES |
|  | VALUE_AS_NUMBER | Numeric value associated with the annotation (e.g., measurement value) | INT | NO |
|  | VALUE_AS_CONCEPT_ID | Concept ID representing the numeric value in the CONCEPT table | INT | NO |
|  | VALUE_SOURCE_VALUE | The source value as it appears in the source data for the annotation | VARCHAR2 (50) | YES |
|  | QUALIFIER_CONCEPT_ID | Concept ID for any qualifier that gives additional context to the annotation | INT | NO |
|  | QUALIFIER_SOURCE_VALUE | The source's specific code or designation for the qualifier | VARCHAR2 (50) | NO |
|  | UNIT_CONCEPT_ID | Concept ID for the unit of measure for any numeric values | INT | NO |
|  | UNIT_SOURCE_VALUE | The source's specific code or designation for the unit of measure | VARCHAR2 (50) | NO |
| **FILEPATH** | FILEPATH_ID | Unique identifier for each file path entry | INT | YES |
|  | FILEPATH_FORMAT_CONCEPT_ID | Concept ID representing the type of file | INT | YES |
|  | FILEPATH | The full file path where the image data is stored | VARCHAR2 (250) | YES |
|  | FILE_NAME | The name of the file without path or extension | VARCHAR2 (50) | YES |
|  | FILE_EXTENSION | The file extension indicating the file type | VARCHAR2 (50) | YES |
|  | FILE_SIZE | The size of the file (BYTE) | INT | NO |
