# Supplementary material for: Integrating Clinical Data and Medical Imaging in Lung Cancer: Feasibility Study Using the Observational Medical Outcomes Partnership Common Data Model Extension
Source: JMIR Med Inform. 2024 Jul 12;12:e59187. doi: 10.2196/59187 (PMC11282389; doi:10.2196/59187)
Supplement: Multimedia Appendix 2 [file medinform_v12i1e59187_app2.docx]

## Appendix 2

Table. I-CDM mapping OMOP CDM Concept ID

| **I-CDM** | | **Concept** | | | | | | **RadLex** |
| --- | --- | --- | --- | --- | --- | --- | --- | --- |
| **Table** | **Column** | **CONCEPT_ID** | | **NAME** | **CLASS** | **DOMAIN** | **VOCAB** | **radiology lexicon** |
| IMAGING_  STUDY | STUDY_TYPE_CONCEPT _ID | 32882 | | Standard algorithm from EHR | Type Concept | Type Concept | Type Concept |  |
|  | MODALITY_CONCEPT_ID | 4300757 | | Computed tomography | Procedure | Procedure | SNOMED | RID10321 (computed tomography) |
|  |  | 4013636 | | Magnetic resonance imaging | Procedure | Procedure | SNOMED | RID10312 (magnetic resonance imaging) |
|  |  | 4056681 | | Plain radiography | Procedure | Procedure | SNOMED | RID10351 (digital radiography) |
| IMAGING_  SERIES | SERIES_ CONCEPT_ID | 2000000464 | | Body Part Examined |  |  | Custom Code |  |
|  |  | VALUE_AS_CONCEPT_ID | 4111459 | Entire lung | Body Structure | Spec Anatomic Site | SNOMED | RID1301 (lung) |
|  |  |  | 4109932 | Entire chest wall | Body Structure | Spec Anatomic Site | SNOMED | RID2468 (chest wall) |
|  |  |  | 4120138 | Entire abdomen | Body Structure | Spec Anatomic Site | SNOMED | RID56 (abdomen) |
|  |  |  | 4121143 | Entire heart | Body Structure | Spec Anatomic Site | SNOMED | RID1385 (heart) |
|  |  |  | 4120136 | Entire neck | Body Structure | Spec Anatomic Site | SNOMED | RID7488 (neck) |
|  |  |  | 4242541 | Entire body as a whole | Body Structure | Spec Anatomic Site | SNOMED | RID39569 (whole body) |
|  |  |  | 4120126 | Entire rib | Body Structure | Spec Anatomic Site | SNOMED | RID2471 (rib) |
|  |  |  | 37118371 | Entire pulmonary blood vessel | Body Structure | Spec Anatomic Site | SNOMED | RID3696 (pulmonary arteritis) |
|  |  |  | 4118108 | Entire thorax | Body Structure | Spec Anatomic Site | SNOMED | RID1243 (thorax) |
|  |  |  | 4025117 | Extremity part | Body Structure | Spec Anatomic Site | SNOMED | RID2638 (lower extremity) |
|  |  |  | 4119359 | Entire brain | Body Structure | Spec Anatomic Site | SNOMED | RID6434 (brain) |
|  |  |  | 4118107 | Entire head | Body Structure | Spec Anatomic Site | SNOMED | RID9080 (head) |
|  |  |  | 4200876 | Iliac spine structure | Body Structure | Spec Anatomic Site | SNOMED | RID38787 (iliac spine) |
|  |  |  | 4186430 | Neuron | Body Structure | Spec Anatomic Site | SNOMED | RID15865 (neural tissue of brain) |
|  |  |  | 4151591 | Base of skull structure | Body Structure | Spec Anatomic Site | SNOMED | RID9196 (skull) |
|  |  |  | 37115364 | Entire bone of spine | Body Structure | Spec Anatomic Site | SNOMED | RID34813 (bone spine) |
|  |  |  | 4082957 | Entire central nervous system | Body Structure | Spec Anatomic Site | SNOMED | RID4025 (central nervous system neoplasm) |
|  |  |  | 4108406 | Entire liver | Body Structure | Spec Anatomic Site | SNOMED | RID58 (liver) |
|  |  |  | 4296995 | Entire elbow region | Body Structure | Spec Anatomic Site | SNOMED | RID2010 (elbow) |
|  |  |  | 4118106 | Entire hip region | Body Structure | Spec Anatomic Site | SNOMED | \|  \| \| --- \|   RID2639 (hip) |
|  |  |  | 4178033 | Entire knee region | Body Structure | Spec Anatomic Site | SNOMED | RID2743 (knee) |
|  |  |  | 4158801 | Entire ankle region | Body Structure | Spec Anatomic Site | SNOMED | RID28545 (ankle) |
|  |  |  | 4041832 | Entire pelvis | Body Structure | Spec Anatomic Site | SNOMED | RID2507 (pelvis) |
|  |  | 2000000465 | | Laterality |  |  | Custom Code | RID5821(Laterality) |
|  |  | VALUE_AS_CONCEPT_ID | 4086896 | Axial | Qualifier Value | Observation | SNOMED | RID10579(axial plane) |
|  |  |  | 4216982 | Coronal | Qualifier Value | Observation | SNOMED | RID10570(coronal plane) |
|  |  |  | 4148957 | Sagittal | Qualifier Value | Observation | SNOMED | RID10574(sagittal plane) |
|  |  | 2000000474 | | BB/NonBB |  |  | Custom Code |  |
|  |  | VALUE_AS_CONCEPT_ID | 45884084 | Positive | Answer | Meas Value | LOINC |  |
|  |  |  | 45878583 | Negative | Answer | Meas Value | LOINC |  |
|  |  | 2000000466 | | Slice Thickness |  |  | Custom Code | RID28669(slice Thickness) |
|  |  | 2000000467 | | Window Center |  |  | Custom Code |  |
|  |  | 2000000468 | | Window Width |  |  | Custom Code |  |
|  |  | 2000000469 | | Patient Position |  |  | Custom Code | RID10420(patient position) |
|  |  | 2000000470 | | Rows |  |  | Custom Code |  |
|  |  | 2000000471 | | Columns |  |  | Custom Code |  |
|  |  | 2000000472 | | Number of instance |  |  | Custom Code |  |
|  |  | 2000000473 | | Series Description |  |  | Custom Code |  |
| IMAGING_  ANNOTATION | ANNOTATION_CONCEPT_ID | 3049878 | | Annotation comment [Interpretation] Narrative | Clinical Observation | Measurement | LOINC |  |
|  |  | 4042996 | | Volume | Qualifier Value | Observation | SNOMED | RID28668 (volume) |
|  |  | 4256609 | | Surface | Qualifier Value | Meas Value | SNOMED |  |
|  |  | 4011459 | | Long axis | Qualifier Value | Observation | SNOMED |  |
|  | QUALIFIER_CON | 4112230 | | First | Qualifier Value | Meas Value | SNOMED | RID5996 (1st) |
|  |  | 4217781 | | Second | Qualifier Value | Meas Value | SNOMED | RID5998(2nd) |
|  |  | 4086896 | | Axial | Qualifier Value | Observation | SNOMED | RID10579(axial plane) |
|  |  | 4216982 | | Coronal | Qualifier Value | Observation | SNOMED | RID10570(coronal plane) |
|  |  | 4148957 | | Sagittal | Qualifier Value | Observation | SNOMED | RID10574(sagittal plane) |
|  | UNIT_CONCEPT_ID | 8588 | | millimeter | Unit | Unit | UCUM |  |
|  |  | 9572 | | square millimeter | Unit | Unit | UCUM |  |
|  |  | 8686 | | cubic millimeter | Unit | Unit | UCUM |  |
| FILEPATH | FILE_FORMAT_CONPCET_ID | 2000000475 | | DICOM image |  |  | Custom Code |  |
|  |  | 2000000476 | | NIfTI |  |  | Custom Code |  |
