# Supplementary material for: Integrating Clinical Data and Medical Imaging in Lung Cancer: Feasibility Study Using the Observational Medical Outcomes Partnership Common Data Model Extension
Source: JMIR Med Inform. 2024 Jul 12;12:e59187. doi: 10.2196/59187 (PMC11282389; doi:10.2196/59187)
Supplement: Multimedia Appendix 3 [file medinform_v12i1e59187_app3.docx]

## Appendix 3

Table. Data Quality Check Rule and Result

| **No** | **CDM_**  **TABLE** | **CONTCEPT_**  **NAME** | **Check description** | **Threshold** | **Result %,**  **(Error N)** |
| --- | --- | --- | --- | --- | --- |
| 1 | IMAGING_STUDY | IMAGING_STUDY_ID | The IMAGING_STUDY_ID must be unique, ensuring each imaging study is distinctively identified within the dataset | No duplicate IMAGING allowed | PASS |
| 2 |  | PERSON_ID | Each PERSON_ID must reference a valid primary key in the PERSON table. All PERSON_ID values should be present in the PERSON table | 100% accuracy in referencing PERSON_ID values | PASS  100 |
| 3 |  | NOTE_ID | Each NOTE_ID must reference a valid primary key in the NOTE table. All NOTE_ID values should be present in the NOTE table | 100% accuracy in referencing NOTE_ID values | PASS  100 |
| 4 |  | PROCEDURE_OCCURRENCE_ID | Each PROCEDURE_OCCURRENCE_ID must reference a valid primary key in the PROCEDURE_OCCURRENCE table. All PROCEDURE_OCCURRENCE_ID values should be present in the PROCEDURE_OCCURRENCE table | 100% accuracy in referencing PROCEDURE_OCCURRENCE _ID values | PASS  100 |
| 5 |  | STUDY_DATE | The STUDY_DATE must be of DATE data type | 100% of DATE data type | PASS  100 |
| 6 |  | FILEPATH_ID | Each FILEPATH_ID must reference a valid primary key in the associated I-CDM table. All FILEPATH_ID values should be present in the corresponding I-CDM table | 100% accuracy in referencing FILEPATH _ID values | PASS  100 |
| 7 |  | STUDY_TYPE_CONCEPT_ID | The STUDY_TYPE_CONCEPT_ID must only contain the concept ID 32882 | 100% of records must have as one of the specified valid ID | PASS  100 |
| 8 |  | MANUFACTURER | MANUFACTURER must exist and be a text value for at least 99% of the records | At least 99% of records must have a non-missing | PASS  99.8,  (287) |
| 9 |  | MODALITY_  CONCEPT_ID | The MODALITY_CONCEPT_ID must contain only one of the following concept IDs 4300757, 4013636, 4056681 | 100% of records must have as one of the specified valid IDs | PASS  100 |
| 10 |  | NUMBER_  OF_SERIES | The NUMBER_OF_SERIES must be equal to the number of series in the IMAGING_SERIES table with the same IMAGING_STUDY_ID. This ensures that the number of series recorded in the IMAGING_STUDY matches the actual series entries in the related table | At least 95% match | PASS  99.9,  (202) |
| 11 |  | NUMBER_  OF_  INSTANCE | The NUMBER_OF_INSTANCE must equal the sum of VALUE_AS_NUMBER for entries in the IMAGING_SERIES table where SERIES_CONCEPT_ID equals NUMBER_OF_INSTANCE, under the condition that they are mapped between the two tables. This is to verify that the number of instances (images) reported in the IMAGING_STUDY corresponds to the aggregated count of instances from the series data | At least 95% match | PASS  99.9,  (109) |
| 12 |  | NUMBER_  OF_SERIES, NUMBER_  OF_  INSTANCE | The presence of a Rule of NUMBER_OF_SERIES necessitates the presence of a Rule of NUMBER_OF_INSTANCE. | At least 95% match | PASS  99.9,  (1) |
| 13 | IMAGING_SERIES | IMAGING_SERIES_ID | The IMAGING_SERIES_ID must be unique, ensuring each imaging study is distinctively identified within the dataset | No duplicate values allowed | PASS |
| 14 |  | PERSON_ID | Each PERSON_ID must reference a valid primary key in the PERSON table. All PERSON_ID values should be present in the PERSON table | 100% accuracy in referencing PERSON_ID values | PASS  100 |
| 15 |  | IMAGING_STUDY_ID | Each IMAGING_STUDY_ID must reference a valid primary key in the IMAGING_STUDY table. All IMAGING_STUDY_ID values should be present in the IMAGING_STUDY table | 100% accuracy in referencing IMAGING_STUDY _ID values | PASS  100 |
| 16 |  | FILEPATH_ID | Each FILEPATH_ID must reference a valid primary key in the associated I-CDM table. All FILEPATH_ID values should be present in the corresponding I-CDM table | 100% accuracy in referencing FILEPATH _ID values | PASS  100 |
| 17 |  | SERIES_DATE | The SERIES_DATE must be of DATE data type | 100% of DATE data type | PASS  100 |
| 18 |  | SERIES_CONCEPT_ID = SliceThickness | VALUE_AS_NUMBER must exist and be a numeric value for at least 99% of the records | At least 99% of records must have a non-missing | PASS  99.8,  (496) |
| 19 |  | SERIES_CONCEPT_ID = Rows | VALUE_AS_NUMBER must exist and be a numeric value for at least 99% of the records | At least 99% of records must have a non-missing | PASS  100 |
| 20 |  |  | Outliers, defined as values beyond the 1st and 99th percentiles, should be reviewed | Outliers should be under 5% | PASS  1.2,  (8,952) |
| 21 |  | SERIES_CONCEPT_ID = Columns | VALUE_AS_NUMBER must exist and be a numeric value for at least 99% of the records | At least 99% of records must have a non-missing | PASS  100 |
| 22 |  |  | Outliers, defined as values beyond the 1st and 99th percentiles, should be reviewed | Outliers should be under 5% | PASS  1.0,  (7,251) |
| 23 |  | SERIES_CONCEPT_ID = BB/NonBB | Values must be exclusively 'Positive' or 'Negative', ensuring they represent these specific states without including the concept IDs 45884084 and 45878583 | 100% of records must have as one of the specified valid IDs | PASS  100 |
| 24 | IMAGING_ANNOTATION | IMAGING_ANNOTATION_ID | The IMAGING_ANNOTATION _ID must be unique, ensuring each imaging study is distinctively identified within the dataset | No duplicate IMAGING_ANNOTATION _ID values allowed | PASS |
| 25 |  | PERSON_ID | Each PERSON_ID must reference a valid primary key in the PERSON table. All PERSON_ID values should be present in the PERSON table | 100% accuracy in referencing PERSON_ID values | PASS  100 |
| 26 |  | FILEPATH_ID | Each FILEPATH_ID must reference a valid primary key in the associated I-CDM table. All FILEPATH_ID values should be present in the corresponding I-CDM table | 100% accuracy in referencing FILEPATH _ID values | PASS  100 |
| 27 |  | IMAGING_STUDY_ID | Each IMAGING_STUDY_ID must reference a valid primary key in the IMAGING_STUDY table. All IMAGING_STUDY_ID values should be present in the IMAGING_STUDY table | 100% accuracy in referencing IMAGING_STUDY _ID values | PASS  100 |
| 28 |  | ANNOTATION_TYPE_CONCEPT_ID | The ANNOTATION_TYPE_CONCEPT_ID must only contain the concept ID 3049878 | 100% of records must have as one of the specified valid ID. | PASS |
| 29 |  | UNIQUE_SERIES_ID | Each UNIQUE_SERIES_ID must reference a valid UNIQUE_SERIES_ID in the IMAGING_SERIES table | 0% error rate | PASS |
| 30 |  | ANNOTATION_DATE | The ANNOTATION_DATE must be of DATE data type | 100% of DATE data type | PASS  100 |
| 31 |  | ANNOTATION_SYSTEM | The ANNOTATION_SYSTEM must contain textual information describing the system of annotation | 100% of records must be non-null | PASS  100 |
| 32 |  | ANNOTATION_METHOD | The ANNOTATION_METHOD must contain textual information describing the method of annotation. | 100% of records must be non-null | PASS  100 |
| 33 |  | ANNOTATION_STATUS | The ANNOTATION_STATUS must contain textual information indicating the status of the annotation | 100% of records must be non-null | PASS  100 |
| 34 |  | ANNOTATION_NUM | The ANNOTATION_NUM must be in a numeric format and cannot be empty. | 100% of records must be numeric and non-null | PASS  100 |
| 35 |  | ANNOTATIO_CONCEPT_ID = Long axis | VALUE_AS_NUMBER must exist and be a numeric value | No missing values for VALUE_AS_NUMBER | PASS  100 |
|  |  |  | Outliers, defined as values beyond the 1st and 99th percentiles, should be identified and reviewed to ensure they accurately reflect the intended measurements. | Outliers should be under 5% | PASS  0.2,  (77) |
| 36 |  | ANNOTATIO_CONCEPT_ID = Volume | VALUE_AS_NUMBER must exist and be a numeric value | 100% of records must be numeric and non-null | PASS  100 |
| 37 |  | ANNOTATIO_CONCEPT_ID = annotation_text | VALUE_SOURCE_VALUE must contain a non-empty text value | 100% of records must be numeric and non-null | PASS  100 |
| 38 |  | ANNOTATIO_CONCEPT_ID = surface area | VALUE_AS_NUMBER must exist and be a numeric value | 100% of records must be numeric and non-null | PASS  100 |
| 39 |  | QUALIFIER_CONCEPT_ID | The QUALIFIER_CONCEPT_ID must contain only one of the following concept IDs 4112230, 4217781, 4086896, 4216982, or 4148957 | 100% of records must have as one of the specified valid IDs. | PASS  100 |
| 40 |  | UNIT_CONCEPT_ID | The UNIT_CONCEPT_ID must contain only one of the following concept IDs8588, 9572, or 8686 | 100% of records must have as one of the specified valid IDs | PASS  100 |
| 41 | FILEPATH | FILEPATH_ID | The FILEPATH _ID must be unique, ensuring each imaging study is distinctively identified within the dataset | No duplicate values allowed | PASS |
| 42 |  | FILEPATH | The FILEPATH field must be in text format and adhere to a valid file path structure | 100% of must conform to the file path format | PASS  100 |
| 43 |  | FILE_NAME | The FILENAME field must be in text format and cannot be empty | 100% of records must be text and non-null | PASS  100 |
| 44 |  | FILE_SIZE | The FILE_SIZE field must contain only numeric values | 100% of records must be numeric | PASS  100 |
