# Supplementary figures and images for: Integrating Clinical Data and Medical Imaging in Lung Cancer: Feasibility Study Using the Observational Medical Outcomes Partnership Common Data Model Extension
Source: JMIR Med Inform. 2024 Jul 12;12:e59187. doi: 10.2196/59187 (PMC11282389; doi:10.2196/59187)

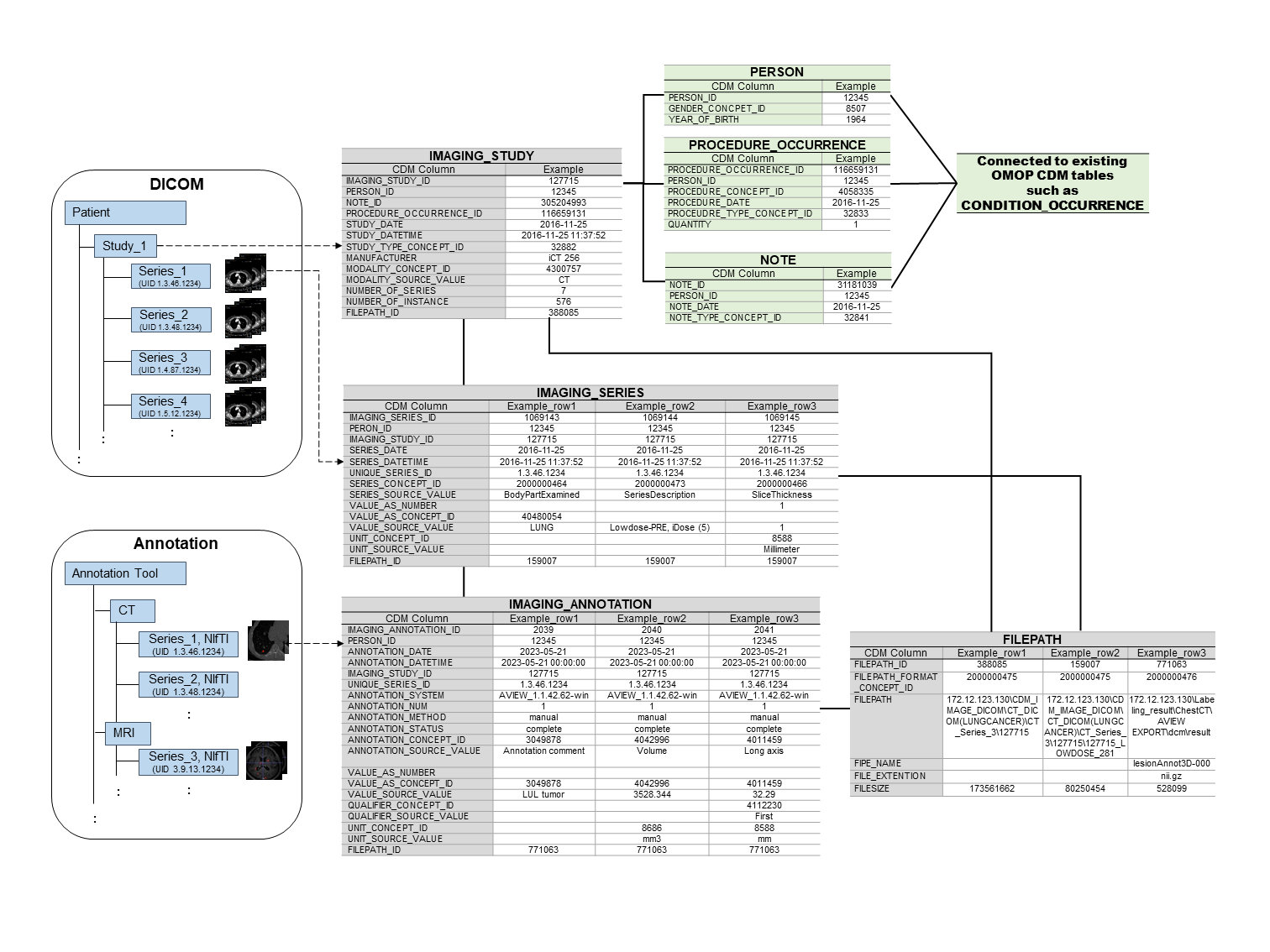

Supplement: Multimedia Appendix 4 [file medinform_v12i1e59187_app4.png]
