# Supplementary material for: Integrating Clinical Data and Medical Imaging in Lung Cancer: Feasibility Study Using the Observational Medical Outcomes Partnership Common Data Model Extension
Source: JMIR Med Inform. 2024 Jul 12;12:e59187. doi: 10.2196/59187 (PMC11282389; doi:10.2196/59187)
Supplement: Multimedia Appendix 5 [file medinform_v12i1e59187_app5.docx]

## Appendix 5

Table. Comparison of MI-CDM and I-CDM

| **Category** | **MI-CDM** | **I-CDM (Our proposal)** |
| --- | --- | --- |
| Extension Table | (1) Image_occurrence (2) Image_feature | (1)Imaging_stduy (2) Imaging_series (3) Imaging_annotation (4) Filepath |
| Data Integration Approach | Connected to OMOP CDM tables and stored image-driven data using existing standard OMOP CDM tables | All image tables are linked to OMOP CDM tables and the extended tables stores image data and annotation data separately |
| Image Access Method | Access images via WADO-RS links | Access images via 'Filepath' table for direct and faster location of images, simplifying integration efforts |
| Customization | Customizable through extension via OMOP CDM's 'measurement' table | Constructed separate image tables using an EAV (Entity-Attribute-Value) structure that allows for the easy addition of necessary data items without altering existing table structures, enabling convenient and effective analysis of image-based data |
| Link to Clinical Information | Linked to 'procedure_occurrence', ’person’, and ’visit_ occurrence’ tables | Linked to 'procedure_occurrence' and ‘person’ tables, particularly connected to the 'note' table to enable efficient analysis of associated radiology reports |
| Imaging Feature | Stores DICOM characteristics in 'measurement' and links to 'image_feature' table for usage | Manages DICOM characteristics directly in an extended image table, highlighting efficiency and intuitive access by presenting essential attributes |
| Standard Terminology Usage | Uses RadLex, LOINC, and SNOMED CT for standardizing terminologies | Prioritizes existing OMOP CDM vocabularies (SNOMED, LOINC, UCUM) and includes additional RadLex mapping information to account for future vocabulary incorporation |
